# *Erythronium japonicum* Alleviates Inflammatory Pain by Inhibiting MAPK Activation and by Suppressing NF-κB Activation via ERK/Nrf2/HO-1 Signaling Pathway

**DOI:** 10.3390/antiox9070626

**Published:** 2020-07-16

**Authors:** Joon Park, Yun Tai Kim

**Affiliations:** 1 Division of Functional Food Research, Korea Food Research Institute, 245, Nongsaengmyeong-ro, Iseo-myeon, Wanju-gun, Jeollabuk-do 55365, Korea; 50029@kfri.re.kr; 2 Department of Food Biotechnology, Korea University of Science and Technology, Daejeon 34113, Korea

**Keywords:** *Erythronium japonicum*, inflammatory pain, neuroinflammation, microglia, Mitogen-activated protein kinase (MAPK), Nuclear factor–kappaB (NF-κB), Nrf2, HO-1

## Abstract

Microglial activation-mediated neuroinflammation influences the development of inflammatory pain. The aim of this study was to investigate the anti-inflammatory effects and mechanisms of aqueous *Erythronium japonicum* extract (EJE) in microglia activation-mediated inflammatory pain. EJE was found to suppress lipopolysaccharide (LPS)-induced inducible nitric oxide synthase (iNOS), cyclooxygenase-2 (COX-2), ionized calcium-binding adapter molecule 1 (IBA-1), and pro-inflammatory cytokines in BV2 microglial cells. In addition, LPS-induced c-Jun NH_2_ terminal protein kinase (JNK) and p38 mitogen-activated protein kinase (MAPK) phosphorylation were inhibited by EJE. Intriguingly, EJE also inhibited p65 phosphorylation by activating extracellular signal-regulated kinase-1/2 (ERK)/nuclear factor erythroid 2-related factor 2 (Nrf2)/heme oxygenase-1 (HO-1) signaling. Furthermore, the effects of EJE treatment, such as HO-1 induction and the reduction of NF-ĸB activation, were reversed by ERK1/2 inhibition. In an inflammatory pain mouse model, Complete Freund’s Adjuvant (CFA)-induced mechanical allodynia and foot swelling were alleviated by the oral administration of EJE. Consistent with in vitro results, EJE increased HO-1, while decreasing CFA-induced COX-2, IBA-1, and pro-inflammatory cytokines in the spinal cord. Among the components of EJE, butanol most heavily suppressed LPS-induced microglial activation and increased HO-1 expression. These findings indicate that EJE can alleviate inflammatory pain by inhibiting p38 and JNK and by suppressing NF-ĸB via ERK/Nrf2/HO-1 signaling.

## 1. Introduction

Inflammatory pain is the perception of harmful stimuli, which occur during inflammatory or immune responses. If the pain persists, the patient’s quality of life is compromised, and further accompanied by adverse side effects, such as emotional discomfort, social damage, and financial loss. In America, the number of patients with chronic pain was recently reported to be in 1 in 5 adults (50 million), generating an enormous economic loss of USD 560 billion each year [1]. Recently, more attention has been focused on developing new therapeutics that control pain using traditional medical herbs and dietary supplements in the field of drug discovery [2,3].

*Erythronium japonicum*, known as the Asian fawn lily, is a pink-flowered trout lily species native to Asia. Traditionally, *Erythronium japonicum* is known to have many medicinal properties and has recently been found to have antioxidant, anti-inflammation, and anti-cancer effects [4,5,6]. However, the analgesic effect of *Erythronium japonicum* is still not understood.

Microglia are the resident macrophage-like cells which play a key role in immune surveillance in the central nervous system (CNS). In response to noxious stimuli or nerve injury, microglial cells become activated and induce inflammatory responses, such as the overexpression of inducible nitric oxide synthase (iNOS) and cyclooxygenase-2 (COX-2) and increased nitric oxide (NO) production. In addition, it secretes abundant pro-inflammatory cytokines, such as Tumor necrosis factor-α (TNF-α), Interleukin-1β (IL-1β), Interleukin–6 (IL-6), and Monocyte chemoattractant protein 1 (MCP-1) [7,8]. A growing body of evidence has shown that neuroinflammation by microglial activation contributes significantly to the development of inflammatory pain [9]. Hence, the inhibition of microglial activation has been suggested as a therapeutic strategy against inflammatory pain.

In the early stages of microglial activation, mitogen-activated protein kinase (MAPK) signaling, which is known to control inflammatory signals, is initiated in response to extracellular stimuli such as lipopolysaccharide (LPS) and pro-inflammatory cytokines [10]. The MAPK pathway consists of c-Jun NH_2_ terminal protein kinase (JNK), p38 mitogen-activated protein kinase (p38), and extracellular signal-regulated kinase-1/2 (ERK 1/2). These three signaling molecules are activated by phosphorylation. Among them, JNK and p38 play an important role in controlling inflammatory responses and cytokine release in microglia. The inhibition of JNK and p38 has been shown to effectively reduce CFA-induced inflammatory pain and microglial activation [11,12]. Therefore, the inhibition of JNK and p38 activation may be key to alleviating inflammatory pain.

Nuclear factor erythroid 2-related factor 2 (Nrf2) is protein that regulates the expression of various protective genes against oxidation and inflammation. Among these protective genes, heme oxygenase-1 (HO-1) plays a critical role in the inhibition of inflammation [13]. In addition, HO-1 is known to exert a protective effect against neuroinflammation. Previous studies have reported that the upregulated phosphorylation of ERK 1/2 may induce the activation of Nrf2, followed by HO-1 expression [14,15,16]. HO-1 has been found to markedly suppress various types of pain by reducing microglial activation [17,18]. Previous research demonstrated that HO-1 decreases Nuclear factor –kappaB (NF-κB) activation by downregulating p65 phosphorylation in the microglia [19]. Furthermore, blocking NF-κB activation alleviated microglial activation and pain allodynia in CFA-induced inflammatory pain [20]. Thus, HO-1 induction is expected to relieve inflammatory pain.

In this study, we evaluated the analgesic effects of *Erythronium japonicum* extract (EJE) and their mechanisms, and observed that EJE acts as an anti-inflammatory analgesic which decreases microglial activation by inhibiting MAPK phosphorylation and by suppressing NF-κB activation via ERK/Nrf2/HO-1 signaling.

## 2. Materials and Methods

### 2.1. Materials

Microglia cells were donated by the Korea Food Research Institute (Wanju, Korea). Chemical reagents were purchased from Sigma-Aldrich (St Louis, MO, USA). RPMI 1640, fetal bovine serum (FBS) and penicillin-streptomycin were obtained from Thermo Scientific Hyclone (Logan, UT, USA). Lipopolysaccharide (LPS) was acquired from Sigma-Aldrich (St Louis, MO, USA). iNOS, COX-2, p-JNK, JNK, p-p38, p38, p-ERK 1/2, ERK 1/2, p-IKKα/β (Ser176/180), IKKβ, p-IκB-α (Ser32), IκBα, p-NF-κB p65 (Ser536), NF-κB p65, HO-1, Nrf2, α-tubulin and Lamin B were purchased from Cell Signaling Biotechnology (Beverly, MA, USA). The antibodies against ionized calcium-binding adapter molecule 1 (IBA-1) and β-actin were obtained from abcam (Cambridge, UK) and Santa Cruz Biotech (Santa Cruz, CA, USA), respectively.

### 2.2. Sample Preparation and Extraction Procedure

Leaves and stems of *Erythronium japonicum* were purchased from Seorak Saradeul (Inje, Korea), and were ground into a fine powder. The powdered samples (500 g) were extracted in reflux apparatus using hot water (5000 mL, at 80 °C) for 6 h. Precipitates were then removed by a vacuum filter. Subsequently, extracts were concentrated into 100 mL with a rotary evaporator. Finally, the samples were freeze-dried to obtain a dry extract, yielding approximately 24.1% of the dried samples, *w*/*w*. The freeze-dried samples were stored in plastic bottles at −20 °C for future use.

### 2.3. Cell Culture and Viability Assay

BV2 cells were grown and maintained in RPMI media supplemented with 5% FBS and 1% penicillin/streptomycin. To assess cell viability, the BV2 cells (5 × 10^4^ cells/mL) were seeded in 96-well plates and incubated at 37 °C in a 5% CO_2_ incubator. After the cells were co-treated with the samples, 20 μL of 3-(4,5-dimethylthiazol-2-yl)-5-(3-carboxymethoxyphenyl)-2-(4-sulfophenyl)-2H-tetrazolium (MTS) reagent (Promega, Madison, WI, USA) was added to each well. After 30 min of incubation, absorbance levels for formazan at 490 and 690 nm were measured using a microplate reader (Bio-Rad Inc., Hercules, CA, USA).

### 2.4. Nitric Oxide (NO) Assay

BV2 cells were seeded into 96-well plates at a density of 5 × 10^4^ cells/well, after which they were incubated for 24 h. Samples were treated at each concentration for 1 h and then treated with LPS (1 μg/mL). The quantity of NO was identified after 24 h. To detect the NO quantity in microglia, cells were incubated with a Griess reagent for 30 min. The absorbance set to 540 nm was measured to detect NO content using a spectrometer reader.

### 2.5. Western Blotting Assay

BV2 cells were washed with Phosphate buffered saline (PBS) and treated with 1× Cell Lysis Buffer (9803S, Cell Signaling Tech) containing 1× protease and phosphatase inhibitor cocktails (78440, Thermo Scientific). After 30 min of incubation on ice, the cells were harvested and centrifuged at 16,000× *g* for 10 min at 4 °C. The supernatant was separated, and protein concentrations were quantified using a BCA protein assay kit (23227, Thermo Scientific). Equal amounts of proteins were separated by SDS-PAGE and transferred onto Polyvinylidene fluoride (PVDF) membranes. After blocking with 5% skim milk in TBST for 1 h at room temperature, the membranes were incubated with primary antibody for overnight at 4 °C. After washing 3 times with TBST, the membranes were incubated with secondary antibody (mouse; A90-116P, rabbit; A120-101P, Bethyl) in a blocking solution for 1 h at room temperature. The protein signals were visualized by ECL Western Blotting Substrate (32106, Thermo Scientific).

### 2.6. Total RNA Isolation and Quantitative Real-Time (qRT-PCR) Assay

Total RNA was isolated using the NucleoPin^®^ RNA XS Kit (Macherey-Nagel, Bethlehem, PA, USA) according to the manufacturer’s instructions. Reverse transcription of RNA was performed with the ReverTra Ace^®^ qPCR RT Master Mix (Toyobo, Osaka, Japan). First-strand cDNA was prepared from 1 μg of total RNA. The real-time PCR reaction was performed in a volume of 20 μL containing 0.1 μg of cDNA, 1 μM of each primer (Table 1), and Power SYBR^®^ Green PCR Master Mix (Applied Biosystems, Carlsbad, CA, USA). Thermal cycling was carried out using a StepOn ePlus Real-Time PCR system (Applied Biosystems) with a program at 95 °C for 5 min., followed by 40 cycles with denaturation at 95 °C for 5 s., and annealing and elongation at 60 °C for 10 s. The gene expression levels were normalized to the expression levels of the GAPDH housekeeping gene. Relative gene expression changes, calculated using the 2-∆∆CT method, are reported as number-fold changes compared to those in the control samples.

### 2.7. Measurement of Pro-Inflammatory Cytokines

BV2 cells (5 × 10^5^ cells/dish) were seeded in 6 cm dishes and cultured for 24 h. Next, the cells were treated with samples at each concentration for 1 h. Subsequently, without washing, the sample-treated cells were incubated with LPS (1 μg/mL) for 24 h. Following the end of the treatment conditions, the culture supernatants were collected to measure the content of pro-inflammatory cytokines. The supernatants were stored at −80 °C until analysis. The concentrations of pro-inflammatory cytokines in the culture supernatant were determined using an ELISA kit (R&D Systems, Inc., Minneapolis, MN, USA) according to the manufacturer’s instructions.

### 2.8. Cytoplasmic and Nuclear Fractionation

BV2 cells (1 × 10^6^ cells/dish) were seeded in 10 cm dishes and incubated at 37 °C for 24 h in a 5% CO_2_ incubator. The medium was replaced with EJE for 1 h. Cells were then treated with 1 μg/mL of LPS and incubated for 4 h. After incubation, cells were collected and washed twice with cold PBS. Nuclear and cytosol protein extractions were prepared with NE-PER Nuclear and Cytoplasmic Extraction Reagents (Thermo Scientific, Rockford, IL, USA) according to the manufacturer’s protocol.

### 2.9. Animals and Experimental Design

All animals received humane care, and the study protocol (KFRI-M-19042) was approved by and performed in accordance with the guidelines for animal use and care of the Korea Food Research Institute. Male C57BL/6 mice (6 weeks old, weight 18–22 g) were purchased from OrientBio (Seong-Nam, Korea). Mice were housed in an air-conditioned room (23 ± 2 °C) with a 12 h light/dark cycle. They were allowed free access to food and tap water. Prior to experimental treatment, all animals were acclimatized for at least 1 week. To establish the inflammatory pain model, CFA (20 μL) was injected into the right hind paw. A total of 50 mice were randomly allocated to each group (*n* = 10 mice per group, 5 groups in total): (i) the control group (Sham), (ii) the group treated with CFA (CFA), (iii) the group co-treated with CFA and 50 mg/kg day EJE (EJE Low; EL), (iv) the group co-treated with CFA and 200 mg/kg day EJE (EJE High; EH), and (v) the group treated with CFA and 10 mg/kg day naproxen (NP). The sham group was used as the control group by injecting the same volume of saline into the right hind paw. After the injection of CFA, EJE and naproxen were orally administered to the mice of the sample treatment groups. During one week, EJE and naproxen were administered daily, and, 1 h later, the mice were evaluated for hyperalgesia. The vertical size of the swelled foot was measured using a caliper (Control Company, Webster, TX, USA).

### 2.10. Von Frey Test to Estimate Mechanical Allodynia

To estimate the effects on pain, mechanical stimulation was carried out to measure the pain threshold before CFA injection (Baseline: BL) and 1, 3, 5, and 7 days after the injection of CFA. For a mechanical hypersensitivity test, mice were placed on a metal mesh floor for 0.5 h in a small acrylic box to adapt to the testing environment. Next, mechanical hypersensitivity was measured using von Frey filaments ranging from 0.02 to 6 g. Filaments were applied perpendicularly to plantar surfaces until the mice responded. If mice did not respond to the initially selected filament, thicker filaments were applied. The minimal value which caused licking, foot lifting, and trembling was recorded as the mechanical withdrawal threshold. After measuring the minimum threshold value, the numbers of responses obtained by stimulating the plantar surfaces of mice 10 times with 0.4 g filaments and cotton were recorded as the withdrawal threshold frequency and cotton swab threshold frequency, respectively.

### 2.11. Extract Fractionation

Solvent fractionation was performed on the extract obtained by hot water extraction. This was extracted with different organic solvents, including hexane (HX), chloroform (CF), ethyl acetate (EA), butanol (BT) and residual water (DW) fractions. The hot water extract (10 g) was suspended in distilled water (100 mL) and initially partitioned with hexane (1:1, *v*/*v*) and then sequentially partitioned with chloroform, ethyl acetate, and butanol to obtain each organic solvent fraction. Finally, residual water fractions were obtained. The fractioning experiment was first conducted at room temperature and the next fractioning experiment was conducted when the previous solvent was completely partitioned. All extracts were concentrated using a rotary evaporator. Finally, dry extracts were obtained through freeze drying. The percentage yields of the HX, CF, EA, BT and DW fractions were 14.4%, 2.4%, 1.6%, 3.1% and 63.2%, respectively. The extract and fractions were stored at −20 °C for future use.

### 2.12. Statistical Analysis

All data were expressed as mean ± SEM and were analyzed by a one-way ANOVA, followed by Dunnett’s post hoc test using Prism 5 (GraphPad Software, Inc., San Diego, CA, USA) for multigroup comparisons. *p* < 0.05 was considered statistically significant.

## 3. Results

### 3.1. EJE Suppresses LPS-Induced Microglial Activation in BV2 Microglia

**Figure 1:**
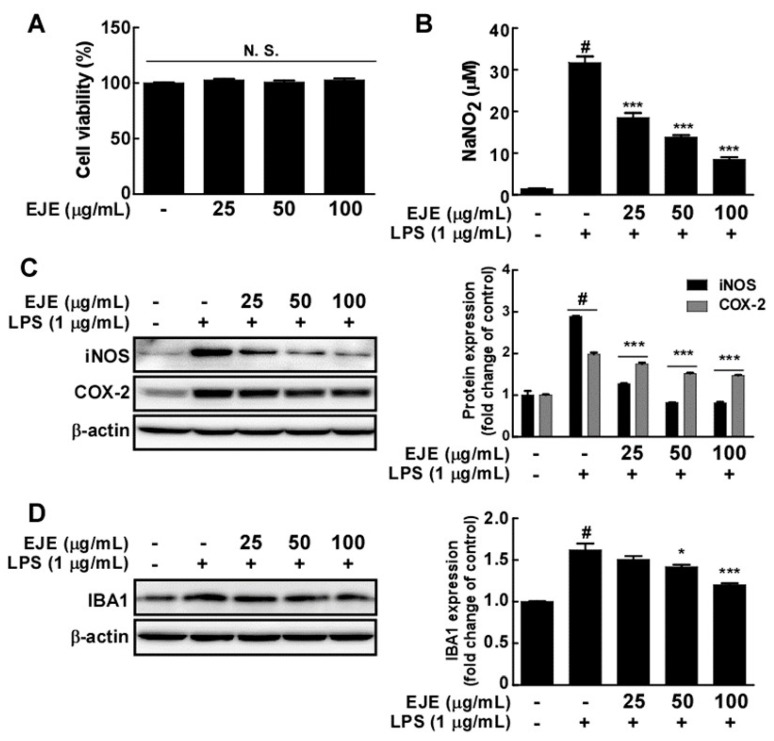
Effect of *Erythronium japonicum* extract (EJE) on lipopolysaccharide (LPS)-induced microglial activation. (**A**) EJE did not affect cell viability in BV2 cells. Cell viability was measured using an MTS assay, as indicated in Materials and Methods. (**B**) EJE inhibited LPS-induced nitric oxide (NO) production in BV2 cells. For the Griess assay, Griess reagents were co-incubated with LPS and EJE-treated culture mediums and measured with a spectrophotometer plate reader, as described in Materials and Methods. (**C**) EJE inhibited LPS-induced inducible nitric oxide synthase (iNOS) and cyclooxygenase-2 (COX-2) expression in BV2 cells. The expression levels of iNOS, COX-2 and β-actin were determined by Western blot assays. (**D**) EJE inhibited LPS-induced ionized calcium-binding adapter molecule 1 (IBA-1) expression in BV2 cells. Expression was detected by Western blotting with a specific antibody. Hash symbols (#) indicate a significant difference (*p* < 0.001) between the control group and the group exposed to LPS alone; asterisks (* and ***) indicate significant differences (*p* < 0.05 and *p* < 0.001, respectively) between groups co-treated with LPS and EJE and the group exposed to LPS alone. In this and all the following Figures, data are presented as the mean ± SEM of three independent experiments.

We first investigated the effects of EJE on the cell viability of BV2 microglia. EJE did not show cell toxicity effects at concentrations ranging from 25 to 100 μg/mL (Figure 1A). Subsequently, we studied the effect of EJE on LPS-induced microglial activation in BV2 microglia. Although LPS treatment induced the overproduction of NO, EJE inhibited LPS-induced NO production in BV2 microglia in a dose-dependent manner (Figure 1B). Furthermore, EJE significantly suppressed LPS-induced iNOS, COX-2 and IBA-1 expression in BV2 microglia (Figure 1C,D).

### 3.2. EJE Inhibits LPS-Induced Pro-Inflammatory Cytokines in BV2 Microglia

**Figure 2:**
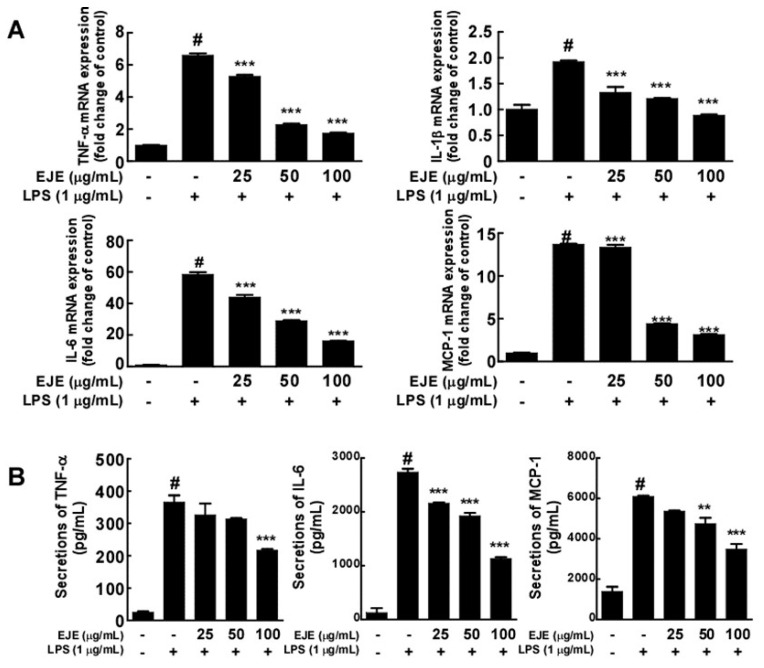
Effect of EJE on LPS-induced mRNA and protein expression of pro-inflammatory cytokines in BV2 cells. (**A**) EJE inhibited LPS-induced *TNF-α, IL-1β, IL-6*, and *MCP-1* mRNA expression in BV2 cells. The production of *TNF-α, IL-1β, IL-6*, and *MCP-1* mRNA was analyzed by quantitative real-time RT-PCR, as described in Materials and Methods. (**B**) EJE inhibited LPS-induced secretion of TNF-α, IL-6 and MCP-1 in BV2 microglial cells. The secretion of TNF-α, IL-6, and MCP-1 was quantified using ELISA kits, as described in Materials and Methods. Hash symbols (#) indicate a significant difference (*p* < 0.001) between the control group and the group exposed to LPS alone; asterisks (** and ***) indicate significant differences (*p* < 0.01 and *p* < 0.001 respectively) between groups co-treated with LPS and EJE and the group exposed to LPS alone. In this and all the following Figures, data are presented as the mean ± SEM of three independent experiments.

We investigated the effect of EJE on the LPS-induced expression and secretion of pro-inflammatory cytokines by BV2 microglial cells. In contrast with treatment with LPS alone, the RT-PCR results revealed that pretreatment with EJE decreased the levels of LPS-induced mRNA of pro-inflammatory cytokines such as TNF-α, IL-1β, IL-6, and MCP-1 (Figure 2A). In addition, EJE inhibited the LPS-induced secretion of TNF-α, IL-6 and MCP-1 in BV2 microglial cells (Figure 2B). However, we did not confirm that EJE reduces LPS-induced IL-1β protein expression in BV2 cells. These results demonstrate that EJE inhibits LPS-induced neuroinflammation.

### 3.3. EJE Inhibits LPS-Induced JNK and p38 Phosphorylation, but Increases ERK1/2 Phosphorylation in BV2 Microglia

**Figure 3:**
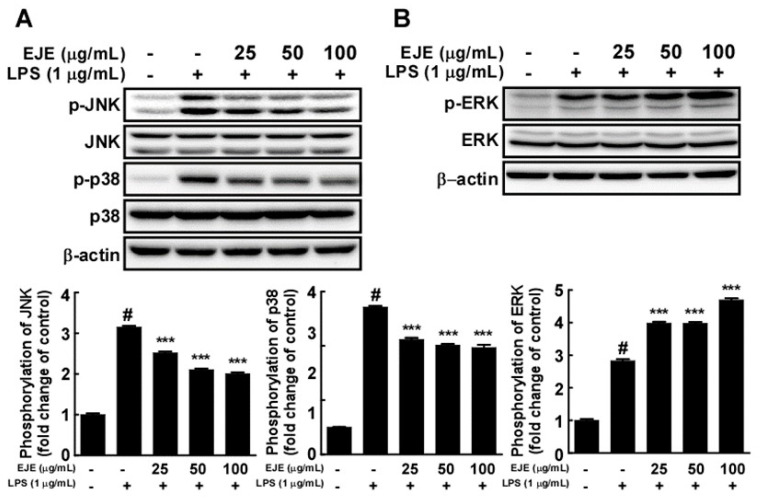
Effect of EJE on the LPS-induced mitogen-activated protein kinase (MAPK) signaling pathway in BV2 cells. (**A**) At 30 min after LPS treatment, EJE inhibited LPS-induced phosphorylation of c-Jun NH_2_ terminal protein kinase (JNK) and p38 mitogen-activated protein kinase (p38) in BV2 microglia. Protein expression was detected by Western blotting with specific antibodies. (**B**) Extracellular signal-regulated kinase (ERK) was more heavily phosphorylated by treatment with EJE. Expression and phosphorylation were detected by Western blot with specific antibodies. Hash symbols (#) indicate a significant difference (*p* < 0.001) between the control group and the group exposed to LPS alone; asterisks (***) indicate significant differences (*p* < 0.001, respectively) between groups co-treated with LPS and EJE and the group exposed to LPS alone. In this and all the following Figures, data are presented as the mean ± SEM of three independent experiments.

To clarify the mechanisms underlying the anti-inflammatory effects of EJE, we assessed the effects of EJE on LPS-induced MAPK phosphorylation in BV2 microglia. Western blotting results revealed that EJE suppressed the LPS-induced phosphorylation of JNK and p38 at 30 min after LPS treatment (Figure 3A). However, treatment with EJE further increased the phosphorylation of ERK 1/2 induced by LPS (Figure 3B). To verify the suppressive effect of EJE on LPS-induced microglial activation, we evaluated whether a JNK inhibitor (SP600125) and a p38 inhibitor (203580) showed similar effects to that of EJE in LPS-treated BV2 microglia. The results show that JNK and p38 inhibition reduced LPS-induced NO production and iNOS as well as COX-2 expression in BV2 microglia (Figure S1A,B). Additionally, JNK and p38 inhibitors had no influence on cell viability at concentrations ranging from 2.5 to 10 μM (Figure S1C). This indicates that the inhibition of JNK and p38 by EJE treatment reduces LPS-induced microglial activation in BV2 cells.

### 3.4. EJE Reduces LPS-Induced NF-ĸB Activation via ERK/Nrf2/HO-1 Signaling in BV2 Microglia

**Figure 4:**
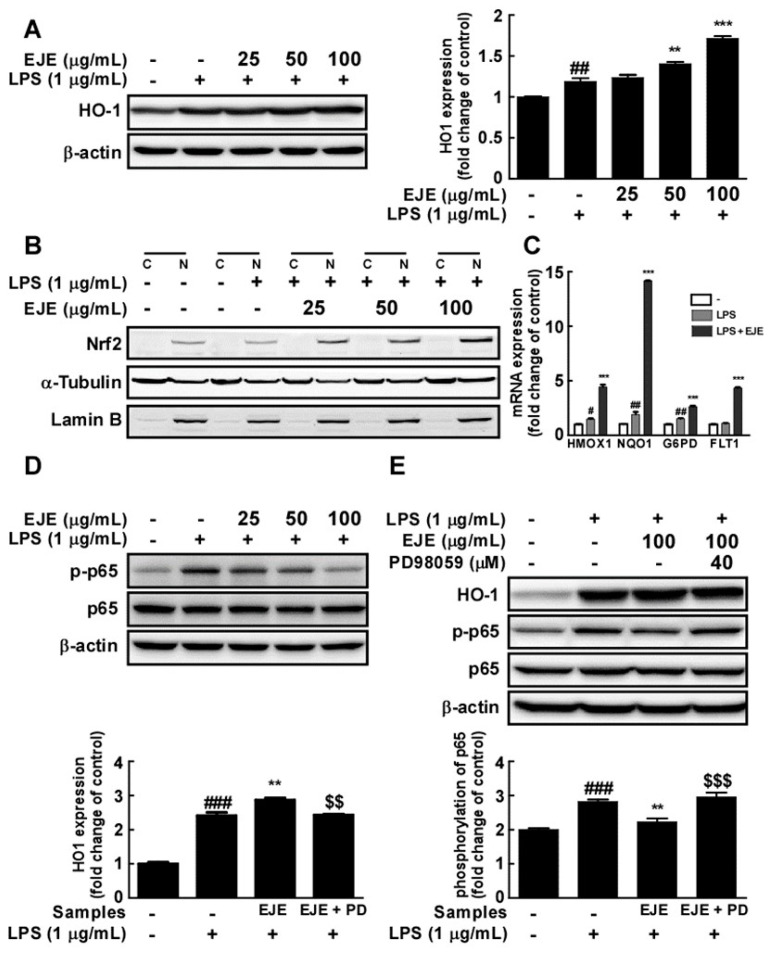
Effect of EJE on LPS-induced heme oxygenase-1 (HO-1) expression and p65 phosphorylation in BV2 cells. (**A**) EJE induced HO-1 expression in BV2 cells. HO-1 expression was confirmed by Western blot. (**B**) EJE induced nuclear factor erythroid 2-related factor 2 Nrf2 expression in the nuclei of BV2 cells. Protein expression was assessed by Western blotting with specific antibodies. (**C**) EJE increased the expression of Nrf2 target genes, including heme oxygenase-1 (*HMOX1*), NAD(P)H quinone dehydrogenase-1 (*NQO1*), glucose-6-phosphate dehydrogenase (*G6PD*) and fms related receptor tyrosine kinase-1 (*FLT1*) in BV2 cells. The expression levels of mRNA were quantified by quantitative real-time RT-PCR, as described in Materials and Methods. (**D**) EJE reduced LPS-induced phosphorylation of p65 at 24 h after LPS treatment. (**E**) ERK 1/2 inhibition reversed the effects of EJE treatment, such as HO-1 induction and the inhibition of LPS-induced p65 phosphorylation in BV2 cells. HO-1 expression and p65 phosphorylation were assessed by each antibody. Hash symbols (#, ## and ###) indicate a significant difference (*p* < 0.05, *p* < 0.01 and *p* < 0.001 respectively) between the control group and the group exposed to LPS alone; asterisks (** and ***) indicate significant differences (*p* < 0.01 and *p* < 0.001 respectively) between the groups co-treated with LPS and EJE and the group exposed to LPS alone; dollar signs ($$ and $$$) indicate significant differences (*p* < 0.01 and *p* < 0.001 respectively) between the groups co-treated with LPS and EJE and the group exposed to LPS, EJE, and PD65059. In this and all the following Figures, data are presented as the mean ± SEM of three independent experiments.

Since EJE treatment increased the phosphorylation of ERK 1/2, we studied whether EJE activated ERK/Nrf2/HO-1 signaling in BV2 microglia cells. EJE administration was followed by significant increases in the expression of HO-1 and Nrf2, as assessed by Western blot of BV2 whole cell extracts (Figure 4A) and cell nuclear extracts, respectively (Figure 4B). EJE also induced the expression of the Nrf2 target genes heme oxygenase-1 (*HMOX1*), NAD(P)H quinone dehydrogenase-1 (*NQO1*), glucose-6-phosphate dehydrogenase (*G6PD*), and fms related receptor tyrosine kinase-1 (*FLT1*) (Figure 4C). To investigate the protective effect of EJE, we confirmed whether EJE inhibited p65 phosphorylation at 24 h after LPS treatment in BV2 microglia. Western blotting results indicated that LPS treatment still induced p65 phosphorylation after 24 h, which was reversed by pretreatment with EJE in BV2 microglia (Figure 4D). In order to prove that these results are related to the upregulated activation of ERK 1/2, we studied whether an ERK 1/2 inhibitor (PD98059) reversed the EJE-treated effects, such as HO-1 induction and the suppression of p65 phosphorylation at 24 h after LPS treatment. As shown in Figure 4E, ERK 1/2 inhibition reversed the EJE-mediated protective effect in BV2 microglia. JNK and p38 inhibitors as well as EJE did not suppress LPS-induced NF-κB signaling at 30 min after LPS treatment (Figure S2A). The results reveal that EJE has a protective effect on LPS-induced NF-κB activation by promoting ERK/Nrf2/HO-1 signaling.

### 3.5. Oral Administration of EJE Alleviates CFA-Induced Pain Hypersensitivity in Mice and Reduces CFA-Induced Microglial Activation and Neuroinflammation in the Spinal Cord of Mice

**Figure 5:**
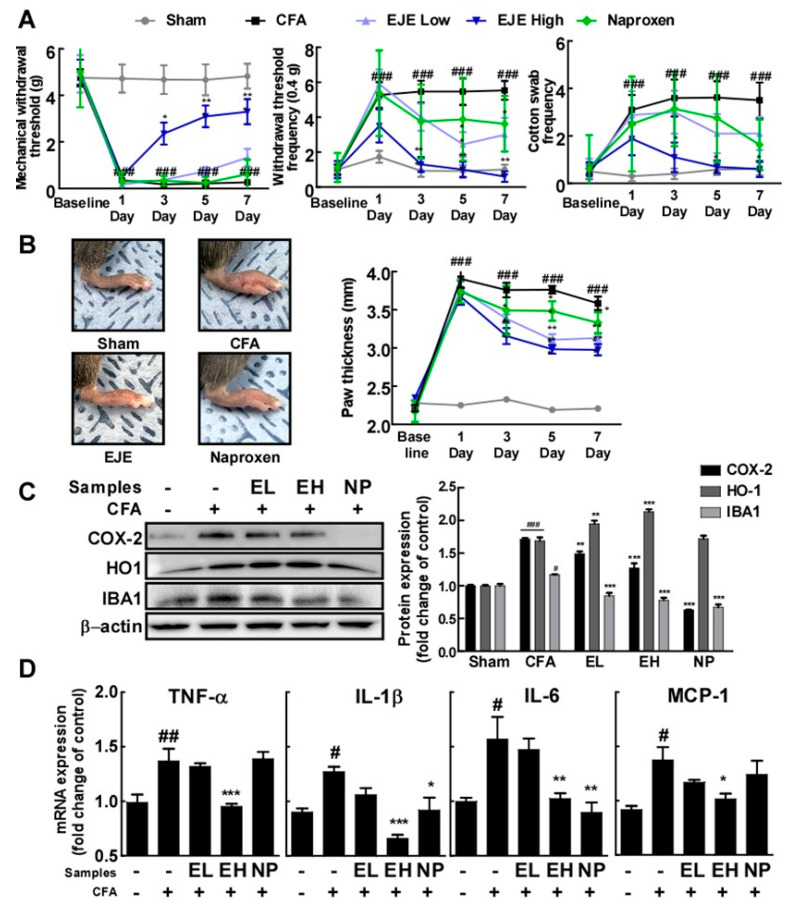
Effect of EJE on Complete Freund’s Adjuvant (CFA)-induced mechanical allodynia in mice and CFA-induced microglial activation and pro-inflammatory cytokine secretion in the spinal cord of 25 mice which were randomly allocated to 5 groups (*n* = 5 mice per group, 5 groups in total): (i) control group (Sham), (ii) group treated with CFA (CFA), (iii) group treated with CFA and 50 mg/kg day EJE (EJE Low; EL), (iv) group treated with CFA and 200 mg/kg day EJE (EJE High; EH), (v) group treated with CFA and 10 mg/kg day naproxen (NP). The sham group was used as a control group in which the same volume of saline was injected into the right hind paw. Naproxen was used as a positive control. (**A**) EJE alleviated CFA-induced mechanical allodynia. (**B**) EJE alleviated CFA-induced foot swelling in mouse. (**C**) EJE reduced CFA-induced COX-2 and IBA-1 expression and increased HO-1 expression. Each expression level was detected by Western blot with antibodies. (**D**) EJE inhibited the secretion of pro-inflammatory cytokines induced by CFA in the spinal cord. The production of *TNF-α, IL-1β, IL-6*, and *MCP-1* mRNA was analyzed by quantitative real-time RT-PCR, as described in Materials and Methods. Hash symbols (#, ## and ###) indicate a significant difference (*p* < 0.05, *p* < 0.01 and *p* < 0.001, respectively) between the control group and the group exposed to LPS alone; asterisks (*, ** and ***) indicate significant differences (*p* < 0.05, *p* < 0.01 and *p* < 0.001, respectively) between groups co-treated with LPS and EJE and the group exposed to LPS alone. In this and all the following Figures, data are presented as the mean ± SEM of three independent experiments.

To estimate the effects of EJE on inflammatory pain, we injected CFA into the hind paws of mice. We used naproxen as a positive control. As shown in Figure 5A,B, the pain behavioral test demonstrated that the intraplantar injection of CFA remarkably reduced the mechanical withdrawal threshold (MWT) and increased the withdrawal threshold frequency (WTF), cotton swab frequency (CSF), and paw thickness. However, the CFA-induced MWT, WTF, CSF, and paw thickness were alleviated by the oral administration of EJE to mice in a dose-dependent manner. These behavioral tests showed that the oral administration of EJE attenuated CFA-induced inflammatory pain in mice. Next, we sought to assess whether oral EJE administration was able to alleviate microglial activation and spinal cord neuroinflammation induced by CFA injection. At 7 days after CFA injection, microglial activation markers were upregulated in the spinal cord (Figure 5C). Nevertheless, the oral administration of EJE reduced the CFA-induced COX-2 and IBA-1 levels in the spinal cord. In addition, EJE upregulated HO-1 expression, consistently with the in vitro results, in the spinal cord. Furthermore, the oral administration of EJE suppressed the secretion of pro-inflammatory cytokines induced by CFA in the spinal cord (Figure 5D). These findings suggest that EJE may inhibit CFA-induced microglial activation.

### 3.6. The Butanol Fraction of EJE Mostly Reduces LPS-Induced Microglial Activation

**Figure 6:**
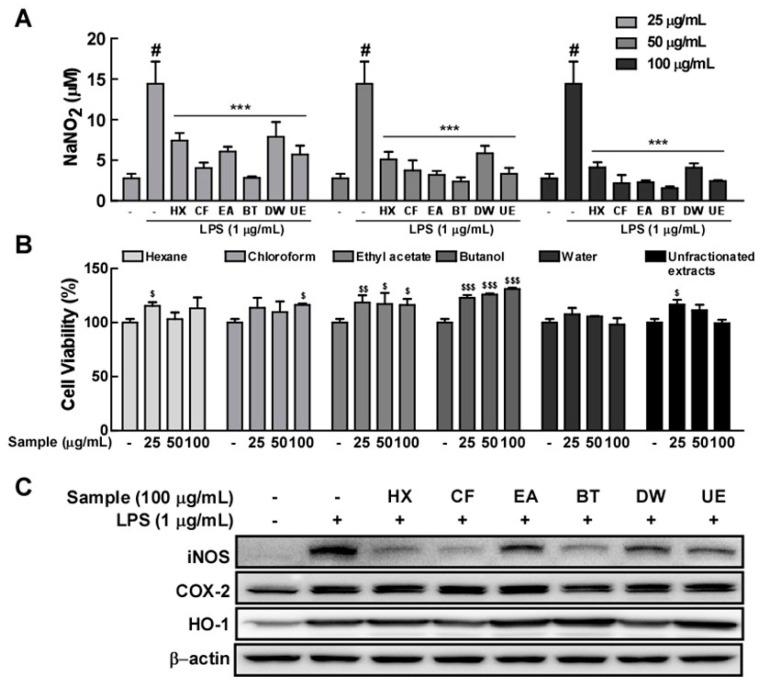
Effect of each component of EJE on LPS-induced NO production and iNOS, COX-2, and HO-1 expression in BV2 cells. EJE was fractionally extracted using the following succession of organic solvents: hexane (HX), chloroform (CF), ethyl acetate (EA), butanol (BT), and residual water (DW) fractions. In this Figure, the unfractionated hot water extract of *Erythronium japonicum* is expressed as unfractionated extract (UE). (**A**) The butanol extracted fraction of EJE was most active among organic solvent extracted fractions in reducing LPS-induced NO production in BV2 cells. NO contents were confirmed by the Griess reagent, as indicated in the Materials and Methods section. (**B**) No fraction of EJE induced any cell toxicity at concentrations ranging from 25 to 100 μg/mL. (**C**) The butanol fraction of EJE decreased LPS-induced iNOS and COX-2 and distinctly induced HO-1 expression in BV2 cells. Expression levels were confirmed by each antibody, as described in Materials and Methods. Hash symbols (#) indicate a significant difference (*p* < 0.001) between the control group and the group exposed to LPS alone; asterisks (***) indicate significant differences (*p* < 0.001, respectively) between the groups co-treated with LPS and samples and the group exposed to LPS alone. In cell viability test, dollar signs ($, $$ and $$$) indicate significant differences (*p* <0.05, *p* < 0.01 and *p* < 0.001, respectively) between the control groups and the groups treated samples. In this and all the following Figures, data are presented as the mean ± SEM of three independent experiments.

To identify the properties of the major components of EJE, a fractionation experiment was conducted. This enabled us to obtain a series of fractions successively extracted in organic solvents, including hexane, chloroform, ethyl acetate, butanol, and H_2_O. Among these fractions of EJE, the butanol fraction substantially reduced LPS-induced NO production in BV2 microglia at concentrations that did not affect cell viability (Figure 6A,B). Additionally, the butanol fraction was the most active amongst the organic extracted phases in causing the reduction of LPS-induced iNOS and COX-2 expression, and strongly increased HO-1 expression (Figure 6C).

## 4. Discussion

In this study, EJE suppressed LPS-induced inflammatory responses and pro-inflammatory cytokines by inhibiting MAPK and NF-ĸB activation in BV2 microglia. Furthermore, the oral administration of EJE alleviated CFA-induced inflammatory pain. Simultaneously, EJE inhibited CFA-induced microglial activation and inflammatory cytokines in the spinal cord. EJE is thus a potential therapeutic strategy to alleviate the inflammatory pain associated with microglial activation.

Microglia-mediated neuroinflammation influences the pathogenesis of pain [21,22]. After being activated in response to various stimuli, activated microglia express inflammatory proteins such as iNOS, COX-2, and IBA-1, which are markers of microglial activation [20,23]. In addition, activated microglia release abundant pro-inflammatory cytokines, including TNF-α, IL-1β, IL-6, and MCP-1, which trigger an neuroinflammatory cascade and considerable damage to the cells and tissues nearby in the CNS [14,20,24]. In this study, exposure to LPS led to the activation of BV2 microglia cells and induced inflammatory responses. However, pre-treatment with EJE inhibited LPS-induced NO production and expression of iNOS, COX-2, and IBA-1. EJE also suppressed the LPS-induced expression of pro-inflammatory cytokines in BV2 microglia. These results demonstrate that EJE inhibited LPS-induced microglial activation and neuroinflammation in BV2 cells.

We next investigated the mechanisms underlying the suppressive effect of EJE in LPS-induced microglial activation. Over the course of microglial activation, the MAPK signaling pathway is activated to induce inflammation and the expression of pro-inflammatory cytokines [25,26]. In our data, LPS treatment induced the activation of the MAPK pathways in BV2 microglia. In MAPK signaling pathways, EJE treatment suppressed the LPS-induced phosphorylation of JNK and p38 in BV2 microglia. Moreover, JNK and p38 inhibitors reduced LPS-induced microglial activation in BV2 cells. Waetzig et al. reported that the inhibition of JNK suppressed the LPS-induced inflammatory cytokines and COX-2 in primary microglia [27]. Additionally, the intrathecal injection of a JNK inhibitor relieved CFA-induced mechanical allodynia in rats [11]. It has recently been reported that p38 plays a critical role in microglial activation and the development of chronic pain [28,29]. It was confirmed that blocking p38 alleviated models of different types of chronic pain by inhibiting microglial activation [30]. It is suggested that inhibiting p38 through EJE effectively contributes to suppressing microglial activation. Therefore, EJE has an inhibitory effect on LPS-induced microglial activation by inhibiting the activation of JNK and p38 in microglia.

In the study underlying anti-inflammatory mechanisms, these results show that EJE inhibited the LPS-induced phosphorylation of JNK and p38 in the MAPK signaling pathway. However, interestingly, it was confirmed that ERK 1/2 was more activated by EJE treatment. Many studies have reported that the activation of ERK 1/2 may initiate the Nrf2/HO1 signaling pathway. These pathways are identified not only in microglia but also in macrophages and endothelial cells, among others [14,31,32,33]. Nrf2 can induce the expression of antioxidant proteins that protect against oxidative stress initiated by inflammation [34,35]. Among the downstream genes induced by Nrf2, HO-1 induction is a representative antioxidant response that protects against inflammatory stimuli. Previous studies have reported that HO-1 has a neuroprotective effect [36] and that HO-1 expression, via Erk/Nrf2 signaling activation, prevents microglial activation in BV2 [14,16,31,33]. Dong Zhao et al. reported that HO-1 induction attenuated LPS-induced NF-κB signaling in microglia [19]. In this study, HO-1 expression was sufficiently induced by pre-treatment with 12-Dehydrogingerdine for 12 h. Our results showed that when LPS was co-treated for 24 h following a 1 h pretreatment with EJE, p65 phosphorylation was suppressed, whereas JNK and p38 inhibitors as well as EJE did not suppress p65 phosphorylation at 30 min after LPS treatment. This suppressive effect seems to be due to the sufficient HO-1 induction by EJE treatment. To confirm the suppressive effect of HO-1 via ERK/Nrf2 signaling induced by EJE, we performed experiments in which ERK 1/2 was inhibited. Intriguingly, ERK 1/2 inhibition reversed the protective effect which induced HO-1 expression and inhibited p65 phosphorylation in BV2 microglia. In this study, we clearly demonstrated that ERK inhibition reversed the induction of HO-1 and suppressed p65 phosphorylation in LPS-treated BV2 microglia. This result suggested that ERK1/2 is upstream of Nrf2/HO-1 signaling, and that activation of the ERK/Nrf2/HO-1 pathway can reduce NF-κB activation. Therefore, EJE inhibited LPS-induced NF-κB signaling by activating the ERK/Nrf2/HO-1 pathway in BV2 microglia.

A previous study has found that a plantar injection of CFA could induce mechanical allodynia via microglial activation and induction of pro-inflammatory cytokines in the spinal cord [20,23,26]. In this study, the oral administration of EJE attenuated CFA-induced mechanical allodynia, microglial activation, and the expression of pro-inflammatory cytokines in the spinal cord. It has been suggested that microglial activation in the spinal cord plays a critical role in the development of chronic pain [37,38]. Meanwhile, inhibiting microglial activation was shown to reduce peripheral inflammation and nerve injury-induced mechanical hypersensitivity [20,39]. Pro-inflammatory cytokines in the spinal cord play an important role in the development and maintenance of chronic pain [9,38,39]. In addition, the intrathecal injection of TNF-α, IL-1β, and IL-6 has been found to significantly induce hypersensitivity [40,41]. Furthermore, blocking pro-inflammatory cytokines via antibodies or knockouts, among other methods, attenuated pain behaviors linked to inflammation or neuropathic pain [42,43,44]. When the nerve is damaged, upregulated MCP-1 attracts macrophages to the nerve. Infiltrated macrophages exacerbate the pain by secreting cytokines that sensitize nerves [45]. Therefore, blocking MCP-1 is also expected to reduce hypersensitivity. These results demonstrate that EJE inhibited CFA-induced mechanical allodynia by blocking microglial activation and pro-inflammatory cytokines.

We performed an extract fractionation to identify the EJE component that most alleviated microglial activation and found it to be the butanol fraction, which suppressed LPS-induced microglial activation and largely increased HO-1 expression in BV2 cells. *Erythronium japonicum* consists of major components, including chlorogenic acid, caffeic acid, kaempferol, quercetin, astragalin, and isoquercitrin [4,46], which have preventive antioxidant and anti-inflammatory effects [16,47,48,49,50]. Among these major components, the evidence has suggested that chlorogenic acid and caffeic acid could attenuate cognitive impairment and neurodegeneration-associated diseases [50]. Hong Yuan et al. found that, in rats, chlorogenic acid was rapidly absorbed and maintained in plasma following oral administration of *Lonicerae japonicae flos* extract containing chlorogenic acid as major component [51]. Additionally, chlorogenic acid was maintained in plasma and entered the CNS following intravenous injection in rats [52]. In a clinical study, individuals who had consumed foods containing caffeic acid showed increased caffeic acids levels in the CNS crossing the blood–brain barrier [53]. However, it remains unclear which bioactive component(s) with anti-microglial activation effects are present in the butanol fraction of EJE. Thus, further investigations are warranted to identify major bioactive components and determine their pharmacodynamics, especially in the CNS.

## 5. Conclusions

In conclusion, this study’s results strongly suggest that EJE significantly suppresses microglial activation. EJE not only reduces JNK and p38 activation, but also inhibits NF-ĸB activation by activating ERK/Nrf2/HO-1 signaling. These double effects of EJE are expected to show stronger alleviative effects on inflammatory pain. Our results suggest that EJE may be a potential candidate to control inflammatory pain.

## Figures and Tables

**Table 1:** Primer sequences for qRT-PCR.

Species	Gene	Primer Sequence (5′-3′)	
		Forward	Reverse
**Mouse**	TNF-α	CCTCTCTCTAATCAGCCCTCTG	GAGGACCTGGGAGTAGATGAG
	IL-1β	ATGATGGCTTATTACAGTGGCAA	GTCGGAGATTCGTAGCTGGA
	IL-6	ACTCACCTCTTCAGAACGAATTG	CCATCTTTGGAAGGTTCAGGTTG
	MCP-1	CAGCCAGATGCAATCAATGCC	TGGAATCCTGAACCCACTTCT
	HMOX1	AAGCCGAGAATGCTGAGTTCA	GCCGTGTAGATATGGTACAAGGA
	G6PD	CACAGTGGACGACATCCGAAA	AGCTACATAGGAATTACGGGCAA
	NQO1	AGGATGGGAGGTACTCGAATC	AGGCGTCCTTCCTTATATGCTA
	FTL1	CCATCTGACCAACCTCCGC	CGCTCAAAGAGATACTCGCC
	GAPDH	GGAGCGAGATCCCTCCAAAAT	GGCTGTTGTCATACTTCTCATGG

## Supplementary Materials

The following are available online at https://www.mdpi.com/2076-3921/9/7/626/s1, Figure S1: Effect of MAPK inhibitors on LPS-induced microglial activation in BV2 cells, Figure S2: Effect of MAPK inhibitors and EJE on LPS-induced NF-κB signaling in BV2 cells.
