# Supplementary material for: Erythronium japonicum Alleviates Inflammatory Pain by Inhibiting MAPK Activation and by Suppressing NF-κB Activation via ERK/Nrf2/HO-1 Signaling Pathway
Source: Antioxidants (Basel). 2020 Jul 16;9(7):626. doi: 10.3390/antiox9070626 (PMC7402134; doi:10.3390/antiox9070626)
Supplement: Supplementary file 1 [file antioxidants-09-00626-s001.pdf]

Supplementary Information for

Erythronium Japonicum Alleviates Inflammatory Pain by Inhibiting MAPK Activation and by Suppressing NF-κB Activation via the ERK/Nrf2/HO-1 Signaling Pathway

Joon Park 1,2 and Yun Tai Kim *

*Corresponding author. Email: ytkim@kfri.re.kr

This file includes:

Fig. S1. Effect of MAPK inhibitors on LPS-induced microglial activation in BV2 cells

Fig. S2. Effect of MAPK inhibitors, EJE on LPS-induced NF-κB signaling in BV2 cells

Supplementary Figure Legend

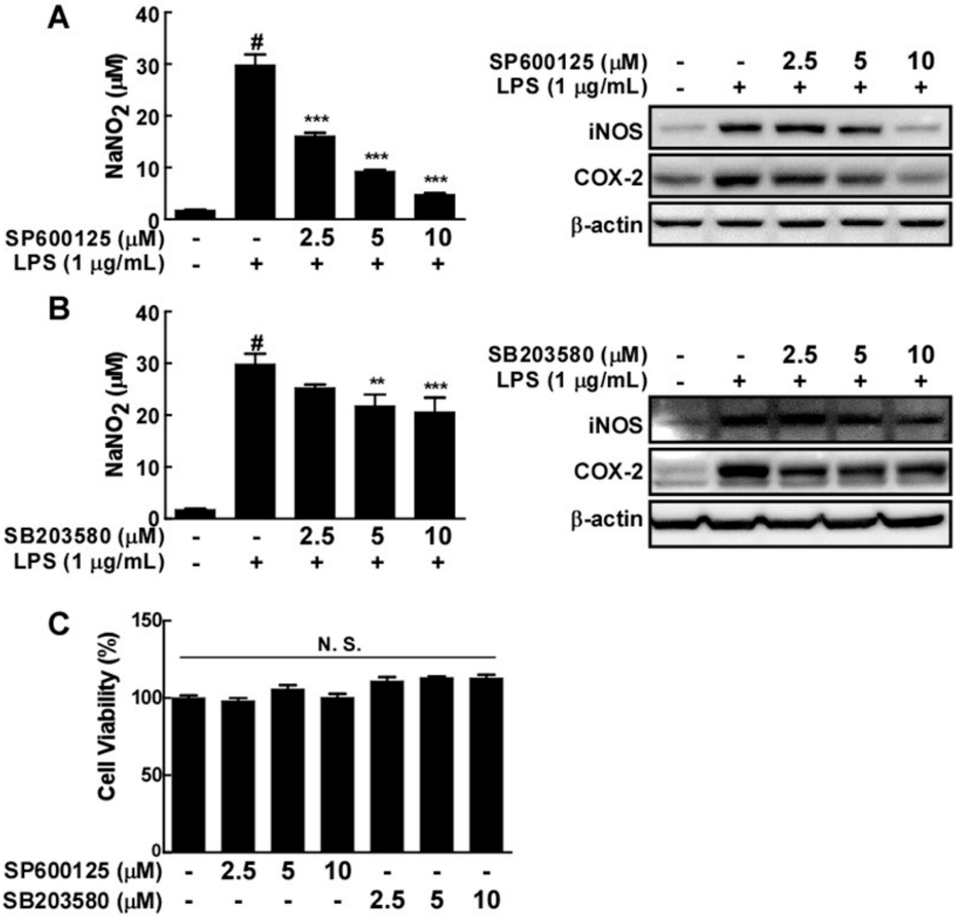

Fig. S1. Effect of MAPK inhibitors on LPS-induced microglial activation in BV2 cells (A) JNK inhibition reduced LPS-induced NO production and iNOS, COX2 expression in dose-dependent manner. (B) p38 inhibition inhibited LPS-induced NO production and iNOS, COX2 expression in dose-dependent manner. (C) The inhibitors didn't affect cell viability. Cell viability were investigated with MTS assay, as indicated in the Materials and Methods. Hash symbols (#) indicate a significant difference ($P < 0.001$) between the control group and the group exposed to LPS alone; Asterisks (** and ***) indicate significant differences ($P <0.01$ and < 0.001, respectively) between groups co-treated LPS and inhibitor and the group exposed to LPS alone. In this and all the following Figures, data are presented as the mean ± S.E.M. of three independent experiments.

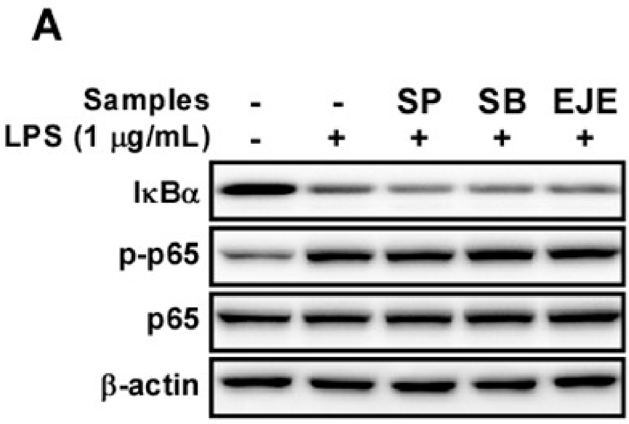

Fig. S2. Effect of MAPK inhibitors, EJE on LPS-induced NF-κB signaling in BV2 cells **(A)** The treatment of JNK, p38 inhibitors and EJE didn`t inhibit LPS-induced NF-κB signaling in BV2 cells at 30 mins after LPS treatment. SP (SP600125) and SB (SB203580) were treated at concentration of 10 μM. EJE was treated in BV2 cells at a concentration of 100 μg/mL. Expression and phosphorylation were detected with each specific antibody.
